# Human Elimination of Organochlorine Pesticides: Blood, Urine, and Sweat Study

**DOI:** 10.1155/2016/1624643

**Published:** 2016-10-05

**Authors:** Stephen J. Genuis, Kevin Lane, Detlef Birkholz

**Affiliations:** ^1^University of Alberta, Edmonton, AB, Canada T6G 2R7; ^2^University of Calgary, Calgary, AB, Canada T2N 4N1; ^3^Department of Chemistry, The King's University, Edmonton, AB, Canada T6B 2H3

## Abstract

*Background*. Many individuals have been exposed to organochlorinated pesticides (OCPs) through food, water, air, dermal exposure, and/or vertical transmission. Due to enterohepatic reabsorption and affinity to adipose tissue, OCPs are not efficiently eliminated from the human body and may accrue in tissues. Many epidemiological studies demonstrate significant exposure-disease relationships suggesting OCPs can alter metabolic function and potentially lead to illness. There is limited study of interventions to facilitate OCP elimination from the human body. This study explored the efficacy of induced perspiration as a means to eliminate OCPs.* Methods*. Blood, urine, and sweat (BUS) were collected from 20 individuals. Analysis of 23 OCPs was performed using dual-column gas chromatography with electron-capture detectors.* Results*. Various OCPs and metabolites, including DDT, DDE, methoxychlor, endrin, and endosulfan sulfate, were excreted into perspiration. Generally, sweat samples showed more frequent OCP detection than serum or urine analysis. Many OCPs were not readily detected in blood testing while still being excreted and identified in sweat. No direct correlation was found among OCP concentrations in the blood, urine, or sweat compartments.* Conclusions*. Sweat analysis may be useful in detecting some accrued OCPs not found in regular serum testing. Induced perspiration may be a viable clinical tool for eliminating some OCPs.

## 1. Introduction

Organochlorinated compounds have been used globally for many years as solvents, fumigants, and insecticides. In the 1930s, Swiss chemist and Nobel Prize recipient Paul Muller first discovered that the most well-known organochlorine agent dichlorodiphenyltrichloroethane (DDT) had significant insecticide properties [1]. Since then, this pest control agent has been utilized around the world to prevent the transmission of many vector-born diseases including malaria and typhus [2]. Alongside DDT, several other organochlorinated pesticides (OCPs) were subsequently developed and were also utilized to control various pests. With unfolding toxicological research, however, evidence of potential human health risks associated with exposure to these agents began to appear. Like many OCPs, it was eventually confirmed that DDT elicited toxic effects that harmed nontarget species, bioaccumulated in the animal food chain, and had a very slow rate of environmental degradation. As a result, the use of DDT was eventually banned in the United States in 1972 [3].

Due to the ability of OCPs to accumulate in body tissues, their long half-life of elimination from the body, and emerging evidence of potential toxicity to human health, many countries throughout the world went on to ban many of the agents within the OCP family [4]. The sequelae of the OCP experiment, however, continue to linger as human contamination with these chemical compounds is still evident throughout the globe. Various factors are contributing to this reality, including the following: (i) the ongoing use of OCPs in some countries, (ii) the persistence of these agents within the environment and within the human body, (iii) the widespread use of international travel resulting in potential OCP exposure when visiting or relocating to jurisdictions where OCPs are still used, (iv) the global exchange of potentially contaminated foodstuffs originating from jurisdictions where OCPs are still used, and (v) the potential for vertical transmission of OCPs from contaminated mothers to offspring during pregnancy and lactation [5]. As a result, OCPs continue to be found in individuals and population groups, including infant children. Individuals contaminated with OCP compounds remain at risk for adverse health consequences and some will potentially pass on these agents to subsequent generations through vertical transmission.

From a clinical perspective, the question remains about how to assess and manage people who have health issues related to xenobiotic contamination [6–8]. As many persistent toxicants including OCPs can sequester and accrue in tissues rather than remaining in the blood compartment [9], biomonitoring for such chemical agents through unprovoked blood and urine analysis does not necessarily reflect the actual body burden of these chemicals [10]. Furthermore, as persistent organochlorine compounds have been associated with myriad health risks, it might be possible to diminish the risk of adverse health sequelae and transgenerational spread of OCPs if means were identified to facilitate elimination of these agents from the human body altogether.

Existing medical literature suggests that elimination of organochlorine compounds and other persistent chemical agents from the human body can have significant clinical benefit in ameliorating health problems [10–12]. Accordingly, the value of establishing interventions to facilitate elimination of toxic chemical agents is thus apparent. In this research study, we provide an overview of literature linking some OCPs with potential human health effects, we endeavor to demonstrate that routine blood testing for OCPs may be inadequate for biomonitoring body burdens, and we provide evidence that transdermal depuration through perspiration facilitates elimination of various OCP compounds.

## 2. Background

OCPs are lipophilic contact insecticides; they have low vapour pressures and slow rates of environmental degradation. These properties make them highly penetrable, long lasting, and extremely effective pesticide agents [13]. These properties also contribute to bioaccumulation within the human organism by the ready absorption into the body and subsequent deposition into adipose tissue. Like medications, the toxicity of OCPs is related to pharmacokinetics, bioavailability, and the molecular topology which is, in turn, related to their molecular size, volatility, and lipophilicity. These factors determine the absorption, distribution, metabolism, excretion, and toxicity (ADMET) of each chemical agent. Additionally, toxicity also depends on the age, nutritional status, and innate detoxification capacity of the host as well as the frequency, intensity, and nature of the toxic exposures [14]. Based on their chemical structures, OCPs can be grouped into 6 categories (Table 1). Similar chemical structures within each group account for the similar chemical properties and comparable ADMET outcomes.

Uptake of OCPs into the human body may occur by ingestion, by inhalation, by vertical transmission, or by transdermal mechanisms. In addition, exposure to OCPs in combination with other pollutants, like inorganic toxic elements, may facilitate an additive or synergistic effect [15]. An interesting study of 702 adults over the age of 70 years, for example, reported significantly higher mortality levels in smokers in the 2nd or 3rd tertile of OCP blood levels but not among smokers in the lowest tertile of exposure [16].

In clinical practice, patients presenting with the sequelae of toxicant exposures are often challenging and misdiagnosed as “they will seldom present with a history of toxic exposure; their symptoms are multisystem and multifactorial; and the findings of the physical exam may provide little confirmation of the intake” [17]. Additionally, there is limited research on the full long-term impact of OCPs and other accrued toxicants within the human body. As a result of the unfolding health problems associated with bioaccumulation of adverse agents [18, 19] and the likelihood of vertical transmission [20] and potential transgenerational impact [21], however, there is emerging attention to the development of effective interventions to facilitate elimination of toxic compounds [6, 22].

To date, it has been realized that organochlorine insecticides exert pathobiological impact including carcinogenicity, neurotoxicity, hormone disruption, and other toxic effects [5, 23–28]. Some studies have linked OCP exposure to significantly higher rates of cancers of the breast, liver, testicles, and lung, as well as sarcoma and non-Hodgkin's lymphoma [26, 29]. OCP exposure has also been linked to higher rates of endometriosis in women [28] and increased risk for diabetes and obesity [30–32], as well as higher risk for neurological disorders like Parkinson's disease [33, 34]. Studies with children and adolescents have linked OCP exposure to neurological and psychiatric sequelae including abnormal reflexes, reduced cognitive development, depression, and behavioral problems [35]. The potential to prevent and/or treat such conditions by identifying individuals with a body burden of these chemical agents, and then treating them to facilitate elimination of their accrued load of toxicants, is worthy of exploration and further research.

### 2.1. Biochemical and Pathophysiological Mechanisms of Harm

Although designed as acute neurotoxins, OCPs have been found to dysregulate human metabolic processes through several different pathways. These pathophysiological mechanisms include mitochondrial damage [24, 36], oxidative stress [37], cell death [38], endocrine disruption [39], and epigenetic modification [40, 41]. As discussed, there is also evidence that concomitant contamination with OCPs along with other toxic chemical agents may exhibit synergistic effects [15].

Additionally, OCPs also exhibit synaptic dysregulation by altering the cation channels (e.g., sodium) at the synapse in nerve cells [42]. Although this effect establishes their efficacy against insect pests, the similarity between organismal synaptic cation channels between species results in nonspecificity and inadvertent harm to nontarget organisms [43]. Another potential pathway of harm is the recently established mechanism entitled “toxicant induced loss of tolerance” (TILT) [44]. This pathophysiologic mechanism resulting in human multimorbidity is characterized by a toxicant burden (e.g., persistent OCPs) resulting in impaired tolerance and hypersensitivity [45]. When triggered by an antigenic incident from the environment (e.g., pollen, chemical agent, or food), an immune response ensues potentially producing antibodies and proinflammatory cytokines. A multisystem response resulting in a condition entitled “Sensitivity Related Illness” (SRI) is often the result [44]. Unfolding research continues to expound on various pathophysiological mechanisms of damage to the human organism.

### 2.2. Human Exposure

There is mounting evidence that individuals and population groups are currently being exposed to a multitude of different types of chemical toxicants, some of which may accrue within the body. The Centers for Disease Control, for example, recently released a study confirming accrual of multiple pollutants in most American adults and children [46]. In a similar nation-wide epidemiological study in Canada, Health Canada also reported on an assortment of stockpiled chemical toxicants within the general population [47].

An issue of particular concern is fetal and early life exposure to xenobiotics at critical times of development as a result of maternal exposure and bioaccumulation [48]. Although OCPs were not specifically measured, a recent cord-blood study revealed an accumulation of various toxicants in neonates at birth [49]. Studies on OCPs within lactating women have confirmed OCP levels in some women that exceed Health Canada's tolerable daily intake (TDI) guidelines [50]. The potential clinical sequelae of such accrual within reproductive-aged women can be seen, for example, in a large observational study examining specific OCP exposures (dicofol and endosulfan) [51]. This research suggests a correlation of increased risk of autistic spectrum disorder based on the distance between maternal residence and the location of pesticide application [51]. Such emerging work on gestational and lactational exposures led to the recent “Special Communication” by the International Federation of Obstetrics and Gynecology [20] (an organization which oversees much of the maternity care throughout the world) in an effort to bring global attention to the reality of widespread vertical transmission of toxicants and the myriad pediatric health problems resulting from maternal xenobiotic pollution.

Although restrictive policies have been implemented for OCP usage in many jurisdictions, these agents continue to be found within individuals and population groups. This is, as discussed, due to continued usage in some areas of the world, vertical transmission to offspring, and persistence within the human organism. Why do they persist? Following exposure, OCP compounds are dechlorinated and conjugated in the liver where biliary excretion is the main mechanism for elimination. However, organochlorine compounds are reabsorbed to some degree in the enterohepatic circulation and this recycling phenomenon accounts for the persistence within the human body [52].

As a result of such persistence and the lipophilic nature of OCPs, these chemical agents tend to store and bioaccumulate in adipose tissues. Although the complete range of toxicity relating to OCP exposure and bioaccumulation is not fully understood and remains somewhat controversial, many adverse sequelae have been linked to exposure to such agents as previously mentioned. Accordingly, interventions are required to assist in facilitating elimination of OCPs, in order to prevent or overcome adverse clinical sequelae resulting from the potential physiological disruption caused by the enduring presence of these toxicants.

Thus far, there has been limited work on interventions to facilitate elimination of OCPs [7, 52–55]. Schnare et al. examined the use of induced perspiration as a means to expedite elimination of PCBs, PBBs, and OCPs [56]. Their work confirmed that enhanced mobilization and excretion via induced perspiration reduced the body burden of hexachlorobenzene (HCB) and 10 polychlorinated biphenyl congeners [57]. Removal of organochlorine compounds has also been facilitated by specific interventions which interrupt the enterohepatic circulation [7, 52, 58]. The main purpose of this study is to determine whether induced perspiration can be used clinically to facilitate decorporation of the range of both parent OCPs as well as their metabolites.

## 3. Materials and Methods

### 3.1. Participant Recruitment

Nine males and 11 females with mean ages of 44.5 ± 14.4 years and 45.6 ± 10.3 years, respectively, were recruited to participate in this study after appropriate ethical approval was received from the Health Research Ethics Board of the University of Alberta. 10 participants were patients with various clinical conditions and 10 were otherwise healthy adults. Participants with health issues were recruited from the first author's clinical practice by invitation and both healthy and sick individuals were selected as samples of convenience by availability, desire to participate, and ease of contact. Each participant in the study provided informed consent and volunteered to give one 200 mL random sample of blood, one sample of first morning urine, and one 100 mL sample of sweat. Demographic and clinical characteristics of all research participants are provided in Table 2.

### 3.2. Samples Collection

All blood samples were collected at one DynaLIFE laboratory site in Edmonton, Alberta, Canada, with vacutainer blood collection equipment (BD Vacutainer, Franklin Lakes, NJ 07417, USA) using 21-gauge stainless steel needles which were screwed into the “BD Vacutainer One-Use Holder” (REF 364815). The 10 mL glass vacutainer was directly inserted into the holder and into the back end of the needle. This process and the use of glass blood collection tubes were used to prevent contamination. Blood was collected directly into plain 10 mL glass vacutainer tubes, allowed to clot, and after 30 minutes were centrifuged for 10 minutes at 2,000 revolutions per minute (RPM). After serum was separated off, samples were picked up by ALS laboratories (about 3 kilometres from the blood collection site) for storage pending analysis. When received at ALS, serum samples were transferred to 4 mL glass vials and stored in a freezer at −20°C, pending transfer to the analytical laboratory.

For urine collection, participants were instructed to collect a first morning midstream urine sample directly into a provided 500 mL glass jar container with Teflon-lined lid on the same day that blood samples were collected. Urine samples were delivered by the participants directly to ALS laboratories, Edmonton. Samples were transferred to 4 mL glass vials and stored in a freezer at −20°C, pending transfer.

For sweat collection, participants were instructed to collect perspiration from any site on their body directly into the provided 500 mL glass jar container with Teflon-lined lid, by placing the jar against their prewashed skin (with toxicant-free soap, water, and nonplastic brush) when actively sweating or by using a stainless steel spatula against their skin to transfer perspiration directly into the glass jar. Stainless steel, made up primarily of iron, chromium, and nickel, was chosen as it is the same material as the needles used in standard blood collections and is reported not to off-gas or leach at room or body temperature. All but one of the participants (19/20) provided 100 mL of sweat. Each of the glass bottles used for sampling in this study was provided by ALS laboratories and had undergone extensive cleaning and rinsing. The containers were deemed appropriate for sweat collection with negligible risk of contamination: laboratory-grade phosphate-free detergent wash; acid rinse; multiple hot and cold deionized water rinses, oven dried, capped, and packed in quality-controlled conditions.

Sweat was collected within 1 week before or after collecting the blood and urine samples. No specifications were given as to how long sweating had commenced before collection. 10 participants collected sweat inside a dry infrared sauna; 7 collected sweat inside a steam sauna, and 3 collected sweat during and immediately after exercise; no specific instruction was given regarding the type or location of exercise. Participants were educated about the research and were asked to meticulously avoid exposure to any potential sources of toxicants around the time of collection. Sweat was delivered by the participants directly to ALS laboratories. Samples were transferred to 4 mL glass vials and stored in a freezer at −20°C, pending analysis. No preservatives were used in the jars provided for sweat and urine collection nor in the serum storage vials.

### 3.3. Laboratory Method Description

Of the 6 categories of OCPs, 5 were analyzed (Table 3, toxaphene was not included in this study). Organochlorine pesticide concentrations of parent and metabolite compounds were analyzed using dual-column gas chromatography with electron-capture detectors (GC-ECD). A pair of surrogate standards, tetrachlorometaxylene (TCMX) and decachlorobiphenyl (DCB), were used to monitor the performance of the method.

The methodology for determining the selected organochlorine pesticides was as follows. Serum samples were weighed into glass tubes (8 g) and 8 mL of methanol was added to the serum samples. Sweat and urine samples were weighed into glass tubes (5 g) and 5 mL of methanol was added to each of the samples. Serial extraction of the bioactive compounds was performed on serum, sweat, and urine samples 3 times by adding 12 mL of an ethyl ether : hexane solution (1 : 1, v/v) and removing the supernatant via centrifugation. The extract was then put through a sodium sulfate column to dry. The resulting extracts were combined and concentrated to 1 mL and put through a 12 g, 2% deactivated florisil column. Florisil was used to eliminate coeluting chlorophenols. External standard calibration was used for quantification. Method blanks were used to ensure quality control, water and calf serum samples. Instrument detection limits were determined to be 0.10 *μ*g/kg. Pentachloronitrobenzene (PCNB) was added to the extracts as an internal standard and the samples were analyzed by dual-column gas chromatography with electron-capture detectors (DB-5 and DB-1701).

## 4. Results and Discussion

The results of the study can be found in Tables 4 and 5. The parent OCP compounds showed differential detection (Tables 4(a)–4(c)) and compounds detected in at least one participant's serum sample include aldrin, DDT, endosulfan I and endosulfan II, heptachlor, mirex, hexachlorobenzene, *α*-HCH, *β*-HCH, and *γ*-HCH. Endrin, *δ*-HCH, and methoxychlor were not detected in any of the participant's serum samples. The parent compounds detected in at least one participant's urine sample include aldrin, DDT, endosulfan I and endosulfan II, endrin, mirex, *γ*- HCH, and methoxychlor. Heptachlor, *α*-HCH, *β*-HCH, *δ*-HCH, and hexachlorobenzene were not detected in any participant's urine sample. All of the parent compounds except the isomers of hexachlorocyclohexane were detected in at least one participant's sweat sample.

The metabolite compounds also showed differential detection. The metabolite compounds detected in at least one participant's serum sample include DDE, endrin ketone, endrin aldehyde, and heptachlor epoxide. The metabolites, DDD, endosulfan sulfate, and dieldrin, were not detected in any serum samples. The metabolite compounds detected in at least one participant's sweat sample include DDE, DDD, endosulfan sulfate, dieldrin, and endrin ketone. Neither endrin aldehyde nor heptachlor epoxide was detected in any sweat samples. The metabolite compounds detected in at least one participant's urine sample include DDD, endosulfan sulfate, dieldrin, endrin aldehyde, and heptachlor epoxide. The metabolites DDE and endrin ketone were not detected in any urine samples.

Of the parent compounds, DDT, methoxychlor, and endrin as well as the metabolites DDE, DDD, and endosulfan sulfate appear to be readily excreted into sweat as they are found in over half of the participants examined (Table 5). Additionally, with the exception of DDE, these OCPs were not readily detected in blood testing while still being excreted and identified in sweat. This may indicate that these OCP compounds are stored and sequestered in tissues, not evident in blood testing, but are mobilized and excreted during perspiration. In contrast, endosulfan I was almost exclusively detected in urine samples of over half of the participants (Table 5). Collectively, the findings suggest that these participants have been commonly exposed to DDT, methoxychlor, endrin, and endosulfan I and are also carrying a body burden of the metabolites DDE, DDD, and endosulfan sulfate.

Most of the compounds examined were detected in less than half of the participants regardless of the matrix (Table 5). This likely reflects a reduced exposure level to these compounds. However, hexachlorobenzene, heptachlor, and endrin ketone were detected in at least 30% of participants and were all predominantly detected in serum samples (Table 5). Although some compounds were detected in participant urine, these levels typically occurred around the limit of detection and at much lower levels than either serum or sweat analysis. With the exception of endosulfan I, this suggests that urine analysis is not reliable as an analytic tool to measure the body burden of these compounds.

The partitioning of these compounds in the body seems to be related to their lipophilicity. With the exception of endosulfan I and its metabolite endosulfan sulfate, all of the compounds detected in over half of the participants have log *K*_ow_ values above 5 (Table 6). This indicates that they are highly lipophilic and fat soluble and are expected to sequester in fat tissue. In comparison to the compounds found predominantly in sweat, hexachlorobenzene and heptachlor have reportedly similar degrees of lipophilicity but were predominantly found in blood (Tables 4(a)–4(c)). Therefore, these findings may suggest that lipophilicity is not the only factor affecting the partitioning of these compounds.

There are limitations to the study and to interpretation of the results. There has been discussion in lay circles that differing means of thermal depuration by varying types of sauna (such as infrared sauna versus steam sauna), exercise, or other perspiration induction activities may result in differences in excretion rates through skin. This study did not have an adequate data set to control for potential differences in the excretion of the range of OCPs between usages of far-infrared sauna, regular sauna, or exercise. Several other issues may impact the reliability and reproducibility of perspiration analysis that were not considered including body site of sampling, timing of collection while perspiring, differences in skin characteristics between individuals, temperature and humidity of the surroundings, and factors such as diet, pharmaceutical intake, and supplement use. Accordingly, attempts to accurately quantify the relative amount of toxicants released into perspiration are limited.

Areas for future research in relation to induced perspiration might include an examination of (i) differences in rates of excretion into perspiration of assorted xenobiotics from diverse sites in the body, (ii) whether there is diurnal variation in the toxicant content of perspiration, (iii) whether hydration status affects the xenobiotic concentration of perspiration, (iv) dermal reabsorption potential of toxicants after perspiration, and (v) the release of xenobiotics in those in fasting states compared to those without caloric restriction.

## 5. Conclusion

As DDT, DDE, DDD, methoxychlor, endosulfan sulfate, and endrin appear to be readily excreted into sweat, induced perspiration appears to be a potential clinical tool to diminish the body burden of these agents. With the exception of DDE, however, these agents are not readily detected in blood testing. This suggests that common blood analysis may not truly represent the body burden of these compounds. As the routine use of unprovoked blood testing may thus be inadequate for biomonitoring body burdens of OCPs, there may be clinical advantages to the induction of perspiration through methods like sauna and/or exercise in order to collect samples for biomonitoring and diagnosis of many retained OCP compounds.

In conclusion, the previous four papers in this “blood, urine, and sweat (BUS)” series have demonstrated that induced perspiration is effective at facilitating the removal of many toxic elements as well as various organic compounds, but not all [59–62]. This OCP study provides evidence that transdermal depuration through perspiration facilitates elimination of some parent and metabolite OCP compounds, but not all. While the absolute amount of each OCP compound released into sweat may be small according to this data, an average adult may sweat more than one liter per hour during exercise. Under thermal stress, maximal rates of sweating may be as high as two to four liters/hour [63]; and sweating rates for “acclimatized” people who regularly use saunas may be as high as two liters/hour [64]. Accordingly, regular sessions of induced perspiration should be considered cumulatively as a potential clinical modality to diminish body burdens of many xenobiotics, including OCP compounds.

## Figures and Tables

**Table 1:** Organochlorine pesticide groupings based on chemical structure.

Group	Constituents
(i) DDT and analogues	DDT DDE DDD Methoxychlor

(ii) Hexachlorobenzene	Hexachlorobenzene

(iii) Hexachlorocyclohexane	*α*-HCH, *β*-HCH, *δ*-HCH, and *γ*-HCH

(iv) Cyclodiene	Endosulfan I and endosulfan II Heptachlor Aldrin Dieldrin Endrin

(v) Chlordecone, Kelevan, and Mirex	Chlordecone, Kelevan, and Mirex

(vi) Toxaphene	Toxaphene

**Table 2:** Participant demographics and general clinical characteristics.

Participant	Gender	Age	Clinical diagnosis	Technique used for sweat collection
1	M	61	Diabetes, obesity, hypertension	Exercise
2	F	40	Rheumatoid arthritis	Steam sauna
3	M	38	Addiction disorder	Steam sauna
4	F	25	Bipolar disorder	Steam sauna
5	F	47	Lymphoma	Steam sauna
6	F	43	Fibromyalgia	Steam sauna
7	F	48	Depression	Steam sauna
8	F	40	Chronic fatigue	Infrared sauna
9	F	68	Diabetes, fatigue, obesity	Steam sauna
10	M	49	Chronic pain, cognitive decline	Exercise
11	M	53	Healthy	Exercise
12	M	23	Healthy	Infrared sauna
13	M	21	Healthy	Infrared sauna
14	F	47	Healthy	Infrared sauna
15	M	53	Healthy	Infrared sauna
16	F	43	Healthy	Infrared sauna
17	F	51	Healthy	Infrared sauna
18	M	46	Healthy	Infrared sauna
19	M	57	Healthy	Infrared sauna
20	F	50	Healthy	Infrared sauna

**Table 3:** Organochlorine pesticides analyzed.

Group	Parent	Metabolite
DDT and analogues	DDT Methoxychlor	DDE, DDD NA

Hexachlorobenzene	Hexachlorobenzene	NA

Hexachlorocyclohexane	*α*-BHC, *β*-BHC, *δ*-BHC, and *γ*-BHC	NA

Cyclodiene	Endosulfan I, endosulfan II	Endosulfan sulfate
	Aldrin	Dieldrin
	Endrin	Endrin ketone Endrin aldehyde
	Heptachlor	Heptachlor epoxide
	*cis*-chlordane, *trans*-chlordane	*trans*-nonachlor

Chlordecone^*∗*^, Kelevan^*∗*^, and Mirex	Mirex	NA

Toxaphene^*∗*^		

^*∗*^Did not test for this specific compound in this study.

**(a):** 

	SE Aldrin	SW Aldrin	U Aldrin	SE DDT	SW DDT	U DDT	SE Endosulfan I	SW Endosulfan I	U Endosulfan I
*n* ^*∗*^	2	5	2	2	14	3	1	1	13
Mean^*∗∗*^	—	0.15	—	—	0.49	—	—	—	0.14
Median^*∗∗*^	—	0.14	—	—	0.42	—	—	—	0.14
Std. Dev.^*∗∗*^	—	0.028	—	—	0.23	—	—	—	0.018
Range	0.17–0.66	0.10–0.17	0.10–0.15	0.22–0.23	0.27–1.1	0.19–0.39	0.11	0.12	0.12–0.18

^*∗*^ *n* represents the number of participants with a detectable amount of the OCP from the 20 total participants examined.

^*∗∗*^For matrices having fewer than 5 individuals with detectable OCP levels, the mean, median, and standard deviation measurements were not provided.

**(b):** 

	SE Endrin	SW Endrin	U Endrin	SE Heptachlor	SW Heptachlor	U Heptachlor	SE Mirex	SW Mirex	U Mirex
*n* ^*∗*^	0	13	1	8	1	0	2	3	2
Mean^*∗∗*^	—	0.18	—	0.37	—	—	—	—	—
Median^*∗∗*^	—	0.15	—	0.33	—	—	—	—	—
Std. Dev.^*∗∗*^	—	0.083	—	0.17	—	—	—	—	—
Range	—	0.11–0.18	0.12	0.15–0.67	0.11	—	0.12–0.18	0.10–0.17	0.11–0.16

^*∗*^ *n* represents the number of participants with a detectable amount of the OCP from the 20 total participants examined.

^*∗∗*^For matrices having fewer than 5 individuals with detectable OCP levels, the mean, median, and standard deviation measurements were not provided.

**(c):** 

	SE Methoxychlor	SW Methoxychlor	U Methoxychlor	SE Hexachlorobenzene	SW Hexachlorobenzene	U Hexachlorobenzene
*n* ^*∗*^	0	16	3	6	1	0
Mean^*∗∗*^	—	0.26	—	0.29	—	—
Median^*∗∗*^	—	0.22	—	0.30	—	—
Std. Dev.^*∗∗*^	—	0.13	—	0.11	—	—
Range	—	0.13–0.70	0.14–0.19	0.18–0.49	0.11	—

^*∗*^ *n* represents the number of participants with a detectable amount of the OCP from the 20 total participants examined.

^*∗∗*^For matrices having fewer than 5 individuals with detectable OCP levels, the mean, median, and standard deviation measurements were not provided.

**Table 5:** Percentage of individuals with detection of organochlorine pesticides within serum, sweat, and urine (*n* = 20).

	Serum	Sweat	Urine
4,4′-DDT	10	70	15
4,4′-DDE	95	70	0
4,4′-DDD	0	65	35
Methoxychlor	0	80	15
Endosulfan I	5	5	65
Endosulfan sulfate	0	85	10
Endrin	0	50	5
Aldrin	10	25	10
Dieldrin	0	15	10
*trans*-nonachlor	0	5	0
BHC (hexachlorobenzene)	30	5	0
*α*-BHC	10	0	0
*β*-BHC	15	0	0
*δ*-BHC	0	0	0
*γ*-BHC	1	0	1
Endosulfan II	10	10	5
Endrin ketone	35	5	0
Endrin aldehyde	5	0	5
Heptachlor epoxide	5	0	15
Heptachlor	40	5	0
*cis*-chlordane	5	5	0
*trans*-chlordane	10	15	10
Mirex	5	15	15

**Table 6:** Octanol/water partition coefficient of key OCPs [65–70].

Compound	log⁡*K*_ow_
Aldrin	6.50
Endrin	5.2
Endosulfan I	3.83
Endosulfate	3.66
*p,p*′-DDT	6.91
*p,p*′-DDE	6.51
*p,p*′-DDD	6.02
Methoxychlor	5.08
Heptachlor	6.10
Hexachlorobenzene	5.73

## Additional Points

*Key Findings*

- DDT and/or its metabolite(s) were found in nearly every participant regardless of age suggesting that exposure is very common.
- Nearly all organochlorine pesticide (OCP) parent compounds and several metabolites were detected in perspiration suggesting that sweating may be efficacious in diminishing the body burden of many of these toxicants.
- There were some parent OCPs, such as endosulfan I and hexachlorobenzene, and some metabolites, such as endrin ketone and heptachlor, that were not readily excreted into perspiration.
- Lipophilicity appeared to be a major factor influencing the efficacy of transdermal OCP elimination but not the exclusive determinant.
- Only endosulfan I appeared to be predominantly detected in urine suggesting that urine analysis has limited value in detection of retained OCPs.
